# Addition of a histone deacetylase inhibitor increases recombinant protein expression in *Medicago truncatula* cell cultures

**DOI:** 10.1038/s41598-017-17006-9

**Published:** 2017-12-01

**Authors:** Rita B. Santos, Ana Sofia Pires, Rita Abranches

**Affiliations:** Plant Cell Biology Laboratory, Instituto de Tecnologia Química e Biológica António Xavier (ITQB NOVA), Av República, 2780-157 Oeiras, Portugal

## Abstract

Plant cell cultures are an attractive platform for the production of recombinant proteins. A major drawback, hindering the establishment of plant cell suspensions as an industrial platform, is the low product yield obtained thus far. Histone acetylation is associated with increased transcription levels, therefore it is expected that the use of histone deacetylase inhibitors would result in an increase in mRNA and protein levels. Here, this hypothesis was tested by adding a histone deacetylase inhibitor, suberanilohydroxamic acid (SAHA), to a cell line of the model legume *Medicago truncatula* expressing a recombinant human protein. Histone deacetylase inhibition by SAHA and histone acetylation levels were studied, and the effect of SAHA on gene expression and recombinant protein levels was assessed by digital PCR. SAHA addition effectively inhibited histone deacetylase activity resulting in increased histone acetylation. Higher levels of transgene expression and accumulation of the associated protein were observed. This is the first report describing histone deacetylase inhibitors as inducers of recombinant protein expression in plant cell suspensions as well as the use of digital PCR in these biological systems. This study paves the way for employing epigenetic strategies to improve the final yields of recombinant proteins produced by plant cell cultures.

## Introduction

Plant cell suspensions are coming to prominence in the Molecular Farming field as a platform for the production of high value molecules. They have several key advantages when compared to conventional production systems, such as the ability to perform post-translational modifications, the use of low cost culture medium and GMP compliance^1^. Unlike microbial cells, plant cells are able to perform the complex glycosylation needed for correct folding and activity of many proteins^2^. Due to cheaper growth conditions, plant cell suspension cultures offer a low-cost alternative to mammalian production systems. In addition, their controlled and confined growth using classical fermentation technology, which allows the implementation of GMP, overcomes regulatory issues concerning the use of transgenic plants (reviewed in^3^).

Molecular Farming has come a long way since 1989, when work by Hiatt and colleagues^4^ was featured on the cover of Nature. Many of the improvements since then were made by transferring knowledge obtained by researchers working on bacterial and mammalian platforms. Although plant cell cultures present many advantageous characteristics, the low final product yields that have been obtained to date remain the main drawback preventing this system from becoming a reliable production platform.

Efforts to reduce the overall cost of production in mammalian cell line systems have focused on increasing the final production yields^5^. One of the strategies used is to supplement media with protein expression inducers such as valproic acid^6,7^, sodium butyrate^8–10^ and hydroxamic acids^11^. These inducers are described to increase recombinant mRNA and protein expression levels by acting as histone deacetylase inhibitors (HDACi). Histone deacetylases (HDACs) remove acetyl groups from histones and their activity is regulated in the cell by the availability of acetyl-CoA (acetyl group donor) and by the HDAC/HAT (histone acetyltransferase) ratio^12^. HDACi are known to block the activity of HDAC enzymes, resulting in the hyperacetylation of histones. In turn, acetylated histones associate less tightly with DNA, facilitating access of the transcriptional machinery and potentially leading to higher mRNA synthesis and protein expression^13^. HDAC enzymes are present from prokaryotes to eukaryotes^14^ and it has been reported that their secondary structure, particularly around the enzyme’s DNA binding site, is highly conserved^15^. Some of these HDACi have recently been used as anti-cancer agents. Suberanilohydroxamic acid (SAHA) has been shown to arrest cancer cell growth, to induce autophagy and apoptosis, and to have an anti-proliferative activity^16,17^. The hydroxamic acid moiety of SAHA binds to the zinc ion located at the end of the catalytic tubular pocket^18^. The insertion of hydroxamic acids inside the catalytic pocket of a HDAC prevents binding of its natural substrates and thus results in inhibition of catalytic activity^18,19^. SAHA is described to inhibit HDAC classes I, II and IV from the RPD3-like superfamily^17^ and was the first HDACi approved by FDA for advanced cutaneous T-cell lymphoma cancer therapy^20^. In plants, these types of compounds have mainly been used for fundamental biology studies (Sodium Butyrate^21,22^, trichostatin A^23–25^ and SAHA, also known as vorinostat^26^) and to our knowledge there is no report of the use of these compounds as inducers of recombinant protein expression in plant cell cultures.

*Medicago truncatula* cell suspensions have been developed recently as a Molecular Farming platform and we have demonstrated a high yield of recombinant protein production for a fungal protein^27^. However, when human recombinant proteins (erythropoietin and lipocalin-type prostaglandin D_2_ synthase) were produced, the same high yields were not observed^28,29^. For this reason, a novel approach to improve the final yields of human recombinant proteins was designed, which involves the use of HDAC inhibitors. With this strategy, we intend to increase transgene expression levels by opening the chromatin through increased histone acetylation.

In this report, the potential of SAHA as a protein expression inducer in *Medicago truncatula* cell suspensions is addressed. Here, it is demonstrated, for the first time, that SAHA is able to increase the levels of expression of the human recombinant lipocalin-type prostaglandin D_2_ synthase (L-PGDS) produced by a stably transformed *M. truncatula* cell line. This protein has been chosen as a model since it is a glycosylated human protein that is involved in several physiological functions. It is localized in a variety of human tissues and is used as a molecular marker (reviewed in^30^). The effect of SAHA on histone acetylation and HDAC inhibition is also investigated.

## Results and Discussion

### Effect of adding SAHA on Medicago cell culture growth

The effect of SAHA on Medicago cell cultures expressing L-PGDS was initially screened by assessing its impact on cell viability. Cell viability was monitored by trypan blue dye exclusion. The addition of SAHA was tested on different days (0, 3 and 7) of growth as well as at different concentrations (2.5 and 5 µM). A cell culture without SAHA was also monitored as a control.

**Figure 1:**
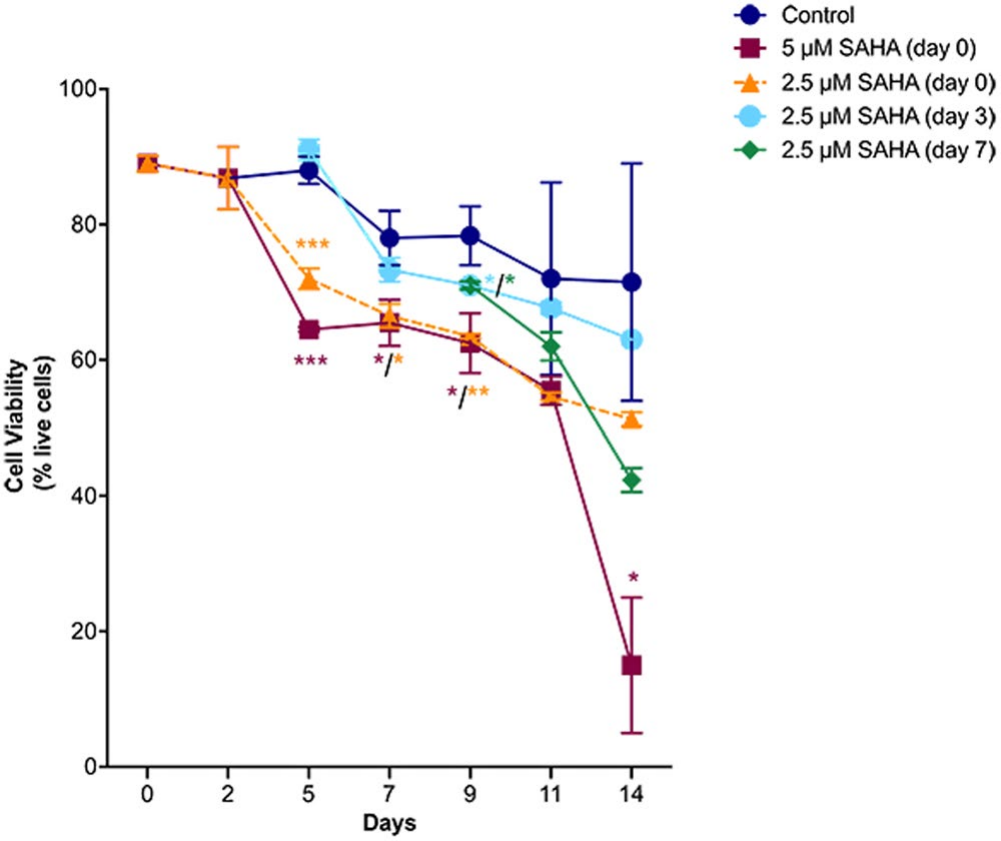
Cell viability tests performed using the trypan blue dye exclusion test throughout 14 days of culture. *p value ≤ 0.05; **p value ≤ 0.01; ***p value ≤ 0.001; statistical analysis represented by *follows the same color code as the graph. Error bars indicate S.E.M. for *n* = 6 (control) and *n* = 3 (SAHA).

When SAHA was added at day 0, at 5 µM final concentration, cell viability dropped from around 90% to 60% on day 5 of growth. By day 14, cell viability decreased to 10%, suggesting that this concentration is unsuitable (Fig. 1). When SAHA was added at a lower concentration (2.5 µM), at day 0, cell viability decreased from 90% to 70% on day 5 of growth, with about 50% viable cells on day 14. Addition of SAHA at the beginning of the experiment appears to have a considerable effect on cell viability and cell division and the results are dose dependent. This might be due to lower cell density during early phases of growth, when compared to SAHA additions later on. The same effect is observed for mammalian cell cultures when this type of compound is used to induce protein expression, due to cell cycle arrest. Usually, inducers of protein expression are only added once a certain cell density has been attained^31^.

Due to the toxic effects observed when using a concentration of 5 µM SAHA, further experiments were performed using a lower concentration (2.5 µM). In order to assess the effect of SAHA on recombinant L-PGDS protein production, two different time points for the addition of SAHA were tested (day 3 and day 7). When added on day 3, corresponding to the onset of exponential phase, SAHA did not have as severe a toxic effect as compared to that observed for addition on day 0 (Fig. 1). In fact, cell viability throughout growth was similar to that observed for the control without SAHA. Moreover, when SAHA was added on day 7 of growth (end of the exponential growth phase), the negative effect on cell viability was far greater than what was observed for addition on day 3. We hypothesize that an addition of SAHA at the end of the exponential growth phase might have a higher toxic effect due to a reduced rate of cell division and associated capacity to metabolize SAHA. Despite the decrease in cell viability following addition on day 7, the resulting protein production and gene expression levels were still assessed.

### Analysis of L-PGDS gene expression

Two independent experiments were performed with SAHA added at different stages of growth; day 3 and day 7. The effect of SAHA on L-PGDS gene expression was analyzed by digital PCR. Digital PCR is a relatively new technique but there are already some reports describing its use in plant studies, namely for GMO quantification^32^ or in disease resistance^33,34^. In this report, digital PCR was chosen to study the expression of recombinant L-PGDS since the treatment with SAHA might affect the expression of any gene, even of the housekeeping genes that are usually used as an internal reference. The use of an internal reference would not be possible and digital PCR allows absolute quantification without the need for an internal control.

The QuantStudio^TM^ 3D (QS3D) AnalysisSuite^TM^ software reads a digital PCR chip and assesses different parameters: the quality of the sample loading, the fluorescent signal from the positive PCR reactions and the background noise present in the chip reading. The chip quality is displayed by flags (green: good quality; yellow: medium quality–needs revision; red: poor quality) as seen on Figure S1. The loading quality is displayed by the continuous green color on the chip (see Figure S1B) and non-amplified and amplified reactions (yellow and blue, respectively) are also represented on chip (Figure S1A) and on scatter plots (Figure S1C).

In Figure S1, digital PCR data for three biological replicates of the same sampling point are shown and the scatter plot depicts the cluster of non-amplified and amplified reactions, which also allows for verification of the homogeneity of the results obtained. The QS3D AnalysisSuite^TM^ software takes into account the dilution factor of the sample in order to give a final value of copies per µL.

**Figure 2:**
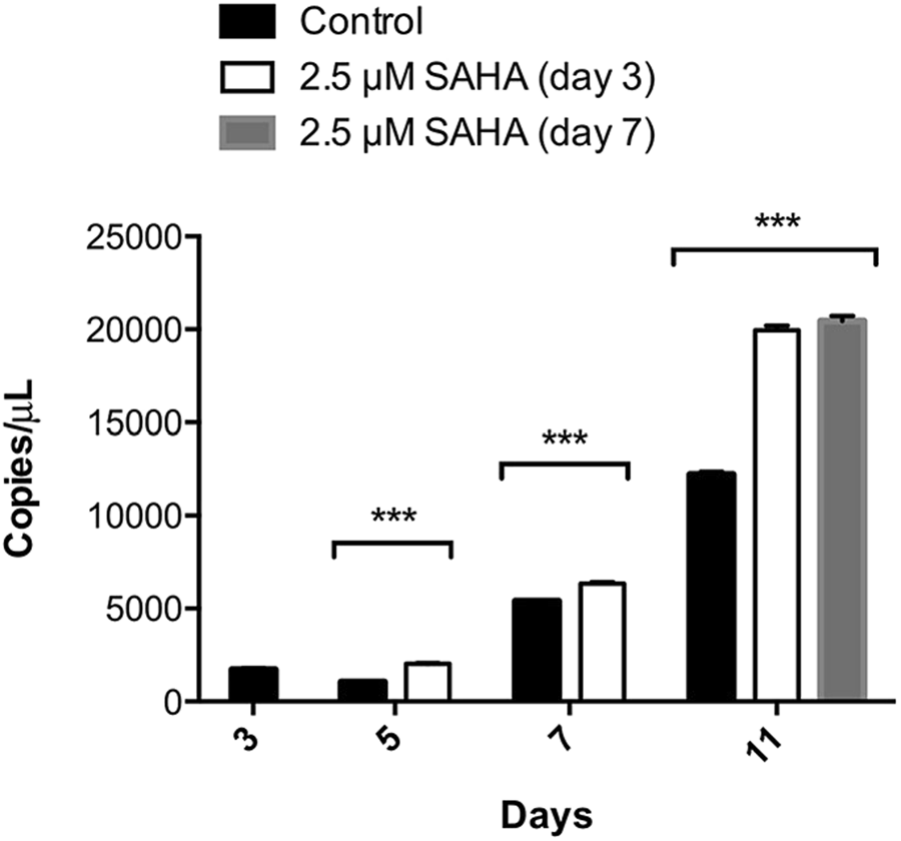
Gene expression analysis of Medicago cell lines expressing L-PGDS, with and without addition of 2.5 µM SAHA (at days 3 or 7 of growth). The calculated sample quantity (Copies/µL) represents the concentration of the cDNA sample in the PCR reaction mix. ***p value ≤ 0.001. Error bars indicate the theoretical confidence interval based on Poisson distribution calculated by the QS3D Analysis Suite^TM^ software for *n* = 6 (control) and *n* = 3 (SAHA).

When SAHA was added, at either day 3 or day 7, the expression of the recombinant L-PGDS gene increased (Fig. 2). At day 11 there was no significant difference in transgene expression between the culture induced on day 3 and that induced on day 7. When compared to the control culture, transgene expression increased 1.9-fold (SAHA addition on day 3) and 2.2-fold (SAHA addition on day 7) for samples taken on day 11 of growth. The increase in gene expression is consistent with the observations made in mammalian cell cultures, where SAHA is considered a protein expression inducer^35^. The recombinant L-PGDS protein expression was thus assessed.

### Screening of recombinant protein levels

**Figure 3:**
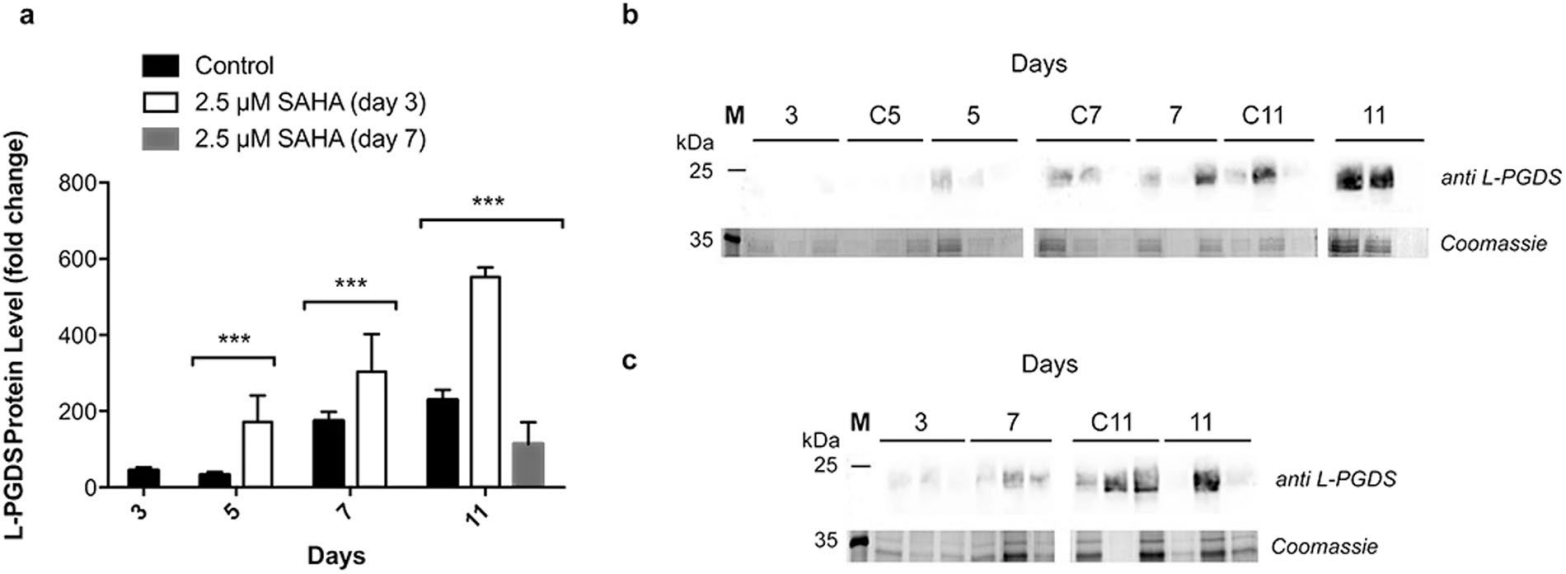
L-PGDS relative protein expression levels in cell medium. (**A**) Relative expression of L-PGDS with and without 2.5 µM SAHA throughout Medicago growth obtained from (**B**) western blot analysis of biological replicates of cell culture samples treated with SAHA on day 3 of growth and (**C**) on day 7 of growth with anti L-PGDS antibody. Control experiment lanes: 3, C5, C7 and C11. **p value ≤ 0.01; ***p value ≤ 0.001. Error bars indicate S.E.M. for *n* = 3. Full-length gels and immunoblots are shown in Supplementary Figure S2.

The production of recombinant L-PGDS, throughout growth, was assessed by western blotting. Addition of SAHA on day 3 resulted in higher accumulation of L-PGDS in the growth medium, compared with the control culture. This higher accumulation was observed two days after addition of the inducer of protein expression, with a 5-fold increase compared to the control (Fig. 3A). Accumulation of L-PGDS protein was also higher for these conditions on day 11, however by this point, the increase was only 2.4-fold when compared to the control. This might be due to degradation of proteins by proteases present in the cell medium (Santos *et al*. in preparation). On the other hand, when SAHA was added on day 7 of growth, a 2-fold decrease in L-PGDS accumulation in Medicago cell medium was observed three days after the addition. We hypothesized that this could be due to intracellular accumulation of L-PGDS protein. In order to assess this possibility, the levels of intracellular L-PGDS were investigated.

**Figure 4:**
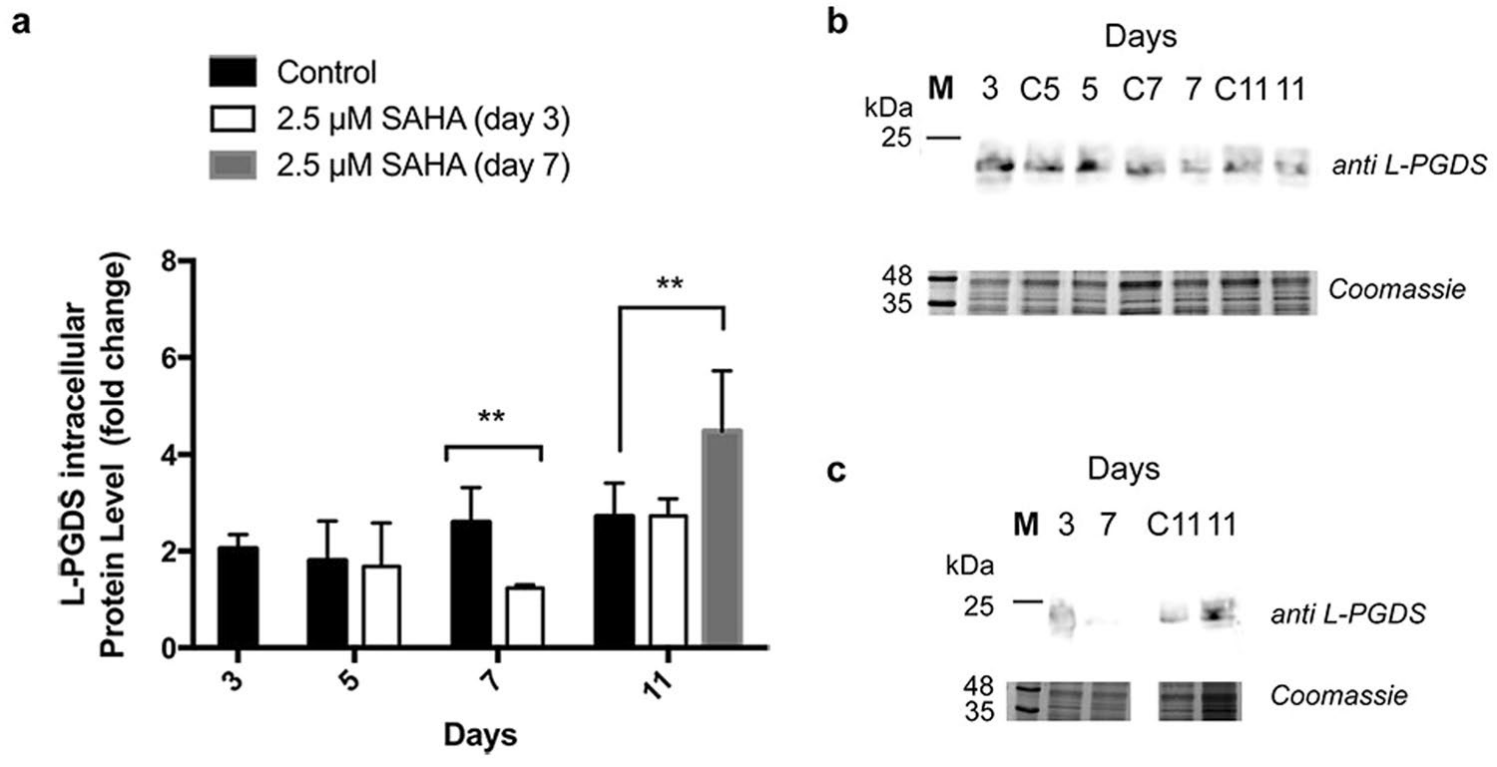
L-PGDS relative protein expression levels in cell extracts. (**A**) Relative expression of L-PGDS with and without 2.5 µM SAHA throughout Medicago cell growth obtained from (**B**) western blot analysis of cell extract samples treated with SAHA on day 3 of growth and (**C**) on day 7 of growth, with anti L-PGDS antibody. Control experiment lanes: 3, C5, C7 and C11. **p value ≤ 0.01. Error bars indicate S.E.M. for *n* = 3. Full-length gels and immunoblots are shown in Supplementary Figure S3.

The amount of intracellular L-PGDS protein detected, when SAHA was added on day 3 of growth, was similar (day 5 and 11) or lower (day 7) than the control (see Fig. 4A and B). Despite a surge in protein production, the recombinant protein follows the secretory pathway, and is mostly released to the culture medium. However, when SAHA was added at day 7 of the growth, the surge in protein production led to the retention of L-PGDS protein inside the cell (see Fig. 4A and C), justifying the low levels of recombinant protein found in the cell medium. This accumulation was unforeseen given that the L-PGDS gene construct possesses a signal peptide that targets the protein to the secretory pathway. It has been described that under conditions of protein production peaks, the endoplasmic reticulum might become overloaded, which can result in greater intracellular accumulation of the recombinant protein^36^. Furthermore, stress or production of defective proteins might lead to unexpected subcellular protein sorting^37^. This phenomenon has already been described in mammalian cell cultures producing recombinant proteins. To overcome this, lower cell incubation temperatures are used to increase the expression of specific proteins. Lower temperatures could increase folding capacity and the expression of endoplasmic reticulum chaperones, which would ensure the recombinant proteins follows the secretory pathway and is exported to the medium^38^. Other studies suggest that the increase of recombinant protein production in CHO cells, following HDACi treatment at 33 °C (a lower temperature than the usually used), can be the result of elevated cellular secretory capacity^39,40^. The combination of these two strategies could be easily implemented in plant cell suspension cultures in order to further increase recombinant protein production.

### Histone deacetylase (HDAC) activity assay

Since transgene expression and recombinant protein levels increased in the presence of 2.5 µM SAHA, an *in vitro* assay was performed in order to confirm that this increase was correlated with HDAC inhibition by SAHA.

**Figure 5:**
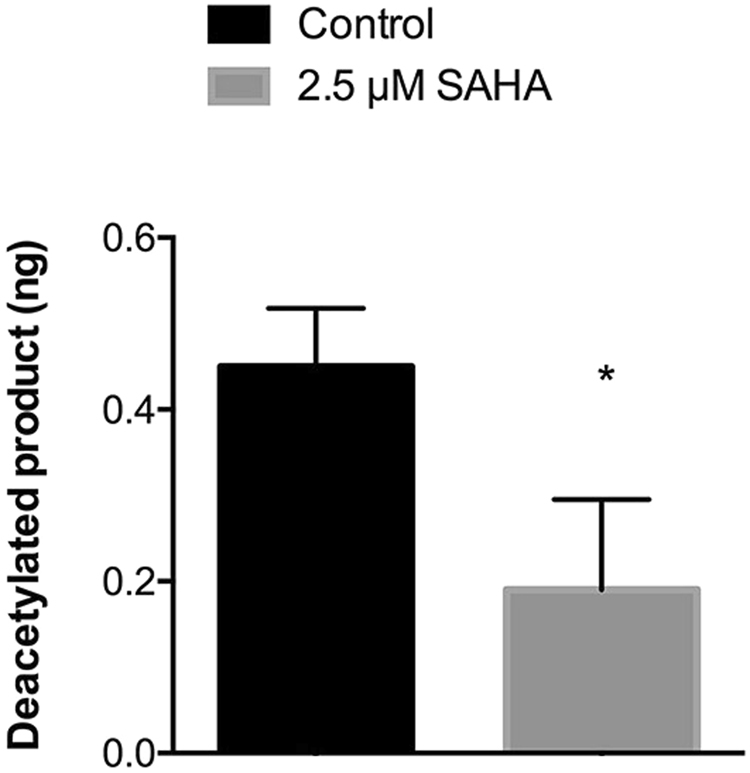
Inhibition of HDAC enzymatic activity. Amount of deacetylated product without and with SAHA. Error bars indicate S.E.M. for *n* = 3. *p value ≤ 0.05.

For this *in vitro* assay, nuclear extracts prepared from cells recovered at day 11 of *M. truncatula* culture were used. It is noteworthy that 2.5 µM SAHA was able to inhibit 74% (calculated using equation (1)) of the HDAC activity in Medicago samples. If HDACs are inhibited, fewer proteins become deacetylated. The amount of deacetylated histone was 2-fold lower in comparison to the control in the presence of SAHA (Fig. 5). This was important to evaluate since there are compounds that are able to inhibit mammalian HDACs but not plant HDACs. For instance, it has been described that sodium butyrate is a HDAC inhibitor in mammalian cells leading to histone hyperacetylation^41^. This compound was tested in alfalfa cells by Waterborg and colleagues, who concluded that it fails to inhibit alfalfa HDACs since it is readily metabolized into acetyl-CoA, which is then incorporated in acetylated histones^42,43^. The results we obtained from *in vitro* experiments confirmed that SAHA is able to inhibit the activity of *M. truncatula* HDACs. However, inhibiting HDACs activity does not correlate directly with histone acetylation since non-histone proteins can also be subjected to their deacetylase activity^44^. Due to this, the level of histone acetylation was also assessed.

### Analysis of histone H3 acetylation levels

The level of histone acetylation was determined by immunoblotting. Cell extracts were recovered along growth after the addition of SAHA at different days and the level of histone H3 acetylation was assessed.

Histone H3 was chosen to study acetylation levels since it has been described as the main target of acetylation in plants, in contrast with mammalian cells and fungi where acetylation is mainly observed on histone H4^43,44^. Since HDACs have different specificity for different lysines^45^, total histone H3 acetylation levels were studied and normalized to histone H3 protein levels.

**Figure 6:**
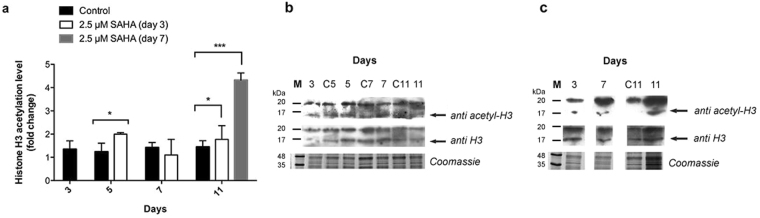
Histone H3 relative acetylation levels. (**A**) Relative acetylation level of histone H3 with and without 2.5 µM SAHA throughout Medicago culture growth obtained from (**B**) western blot analysis of cell extract samples treated with SAHA on day 3 of growth and (**C**) on day 7 of growth, with anti-acetyl-histone H3 and anti-histone H3 antibodies. Control experiment lanes: 3, C5, C7 and C11. *p value ≤ 0.05; **p value ≤ 0.01; ***p value ≤ 0.001. Error bars indicate S.E.M. for *n* = 3. Full-length gels and immunoblots are shown in Supplementary Figure S4.

An increase of histone H3 acetylation levels was observed 2 to 3 days after addition of SAHA in both experiments. When SAHA was added on day 3 of growth, a 1.6-fold increase of acetylation was observed. This increase was maintained throughout growth, with a 1.4-fold increase of acetylation levels observed on day 11 (see Fig. 6A). Addition of SAHA on the 7^th^ day of growth resulted in a 3.1-fold increase of acetylation levels on day 11 (see Fig. 6A).

The analysis of histone H3 acetylation levels confirms that SAHA is able to inhibit HDAC activity *in vitro* (Fig. 5) and this inhibition leads to the accumulation of acetyl groups in histone H3 lysines.

## Conclusion

This report describes for the first time the use of a histone deacetylase inhibitor for the improvement of recombinant protein production in plant cell suspension cultures.

The effect of SAHA addition to *Medicago truncatula* cell culture was assessed at different growth stages. Although some degree of cell toxicity was observed, in a dose dependent manner, SAHA addition at day 3 and day 7 resulted in an increase of recombinant protein accumulation. When SAHA was added on day 7, protein was found to accumulate inside of the cells, rather than being secreted to the medium. This diversion from the targeted destination can result from an ER stress response, triggered by the synthesis of high amounts of recombinant proteins, and can lead to unexpected intracellular protein sorting^46,47^.

This report also demonstrates for the first time, to our knowledge, the use of digital PCR with plant cell culture samples. With this technique, it was possible to observe that addition of SAHA had a stimulatory effect on L-PGDS transgene expression and associated protein production, resulting in the accumulation of protein up to 5-fold higher than the control experiment. To prove the hypothesis that the addition of SAHA increases L-PGDS protein production by inhibiting HDACs and, therefore, increasing histone acetylation levels, the percentage of HDAC inhibition by SAHA was assessed. It was demonstrated for the first time that SAHA inhibits the activity of *Medicago truncatula* HDACs, which results in increased Histone H3 acetylation. Furthermore, the increase of histone H3 acetylation is concurrent with an increase in gene expression and of protein accumulation.

This work lays the first stone for the use of epigenetic modulation as a biotechnology tool to increase the yield of recombinant protein production in plant cell suspension cultures.

## Material and Methods

### Plant material, growth conditions and treatment with HDAC inhibitor

*Medicago truncatula* cell suspension cultures producing human lipocalin-type prostaglandin D_2_ synthase (L-PGDS)^28^ were grown in Murashige and Skoog medium (Duchefa, The Netherlands) supplemented with 30 g/L sucrose (Duchefa, The Netherlands), 4.65 μM kinetin (Duchefa, The Netherlands), 4.52 μM 2.4-D (Sigma-Aldrich, USA) and 65 μM dithiothreitol (Sigma-Aldrich, USA) at pH 5.8, in an orbital shaker (24 °C, 125 rpm) in the dark.

Experiments were established starting by subculturing a two-week old culture. Then, different SAHA concentrations were added at different time points (day 0, 3 and 7) of the culture growth and 2.5 μM SAHA was used for the following experiments. SAHA was added and for each time point biologic triplicates were prepared. SAHA solution was prepared in DMSO (Sigma-Aldrich, USA), which we previously verified did not affect cell growth. Spent medium was collected by vacuum filtration on days 3, 5, 7 and 11 from cultures with and without SAHA and stored at −20 °C.

### Cell viability tests

Cell viability was assessed by dye exclusion (indicative of an intact membrane) using 0.4% trypan blue in PBS-T (Sigma-Aldrich, USA) (1 volume of cells: 1 volume of trypan blue). Live cells with intact membranes are distinguished by their ability to exclude dyes that easily penetrate dead or damaged cells. For each experiment, biologic replicates of all time points were analyzed (six for control experiments and three for SAHA experiments) and the ratio between live and total cells was determined by hemacytomer cell count distributions. A minimum of 50 cells were obtained for cell counting of each replicate. The average percentage of live cells and S.E.M. were calculated.

### Screening of recombinant L-PGDS production

Western blot analysis was performed with spent medium and cell extract samples collected during cell culture growth. Medium samples were 10-fold concentrated using centrifugal filtration units with 10 kDa cut-off Amicon Ultra-15 (Millipore, USA). Cells were ground using liquid nitrogen and a mortar. Extraction buffer (100 mM ascorbic acid, 500 mM NaCl, 5 mM β-mercaptoethanol, pH 8.0) was added to ground cells in 1:1 ratio (g of cells: mL of extraction buffer). Samples were resolved in a 12.5% SDS-PAGE gel and proteins were transferred to a nitrocellulose membrane (0.2 µm, Amersham, UK). The membrane was blocked with 5% skimmed milk powder (Nestlé, Switzerland) and 3% BSA (NZYTech, Portugal) in PBS-T, for 1 hour at room temperature with gentle shaking, followed by incubation with 1:500 anti-L-PGDS (Ab 61866, Abcam, UK) in PBS-T, overnight at 4 °C. Then, the membrane was incubated with 1:8000 HRP conjugated anti-rabbit (A0545, Sigma-Aldrich, USA) in PBS-T, for 2 hours at room temperature with gentle shaking. Signal was detected by chemiluminescence using Amersham ECL Prime reagent (GE Healthcare Life Sciences, UK) in ChemiDoc™ XRS + System (Bio-Rad, USA). Three biological replicates were analyzed and western blots repeated three times.

### Analysis of Histone H3 acetylation levels

Cell extracts were taken from cells recovered by vacuum filtration at day 3, 5, 7 and 11 of growth, as already described. Total protein extracts were resolved in a 15% SDS-PAGE gel and proteins were transferred to a nitrocellulose membrane (0.2 µm, Amersham, UK). The membrane was blocked with 5% BSA (NZYTech, Portugal) in PBS-T for one hour at room temperature, with gentle shaking, followed by an overnight incubation with 1:5000 anti-acetyl-histone H3 (06–599, Merck Millipore, USA) in PBS-T, at 4 °C with gentle shaking. Then, the membrane was incubated in 1:8000 anti-rabbit HRP conjugated (A0545, Sigma-Aldrich, USA) for two hours in agitation at room temperature. Acetyl-histone H3 bands were detected using Amersham ECL Prime reagent (GE Healthcare Life Sciences, UK) and Hyperfilm ECL (GE Healthcare Life Sciences, UK) in a dark room. Then, the membrane was stripped, by washing five times with PBS-T buffer for 30 minutes each. Before reprobing with anti-histone H3 antibody, the membrane was incubated with the secondary antibody anti-rabbit-HRP in order to ensure that the stripping procedure was correctly performed. If no signal was detected, the membrane was washed three times for 5 minutes each with PBS-T and incubated with 1:25000 anti-histone H3 (07–690, Merck Millipore, USA) in PBS-T, at 4 °C, overnight, with gentle shaking. Following incubation for two hours in agitation at room temperature with 1:8000 anti-rabbit HRP conjugated, histone H3 was detected as already described for acetyl-histone H3. Western blots were repeated three times.

Relative quantification of recombinant L-PGDS and levels of acetylated histone H3 was performed using FIJI software^48^ based on the relative intensity of bands^49^.

Immunoblots of L-PGDS, acetylated histone H3 and histone H3 were performed using the protocol described in^50^ in which several SDS-PAGE gel strips are organized in a single nitrocellulose membrane. This allows a correct quantitative comparison between samples on different gels or immunoblots by simultaneous transfer and detection of several samples in the same membrane.

### Nuclei isolation and extraction

Control Medicago cells were harvested by vacuum filtration at day 11 of growth as described above. Cells were ground with liquid nitrogen in a mortar. Ground cells were homogenized with cytoplasmic extraction buffer (25 mM Tris-HCl pH 6.5, 0.45 M sucrose, 5 mM MgCl_2_, 5 mM β-mercaptoethanol, 0.5 mM PMSF, 0.364 µM Pepstatin A, 1.2 µM E64, 0.1% Triton X-100) in a 1:4 ratio (g of cells: mL of cytoplasmic extraction buffer). The homogenate was filtered with a nylon mesh, followed by several filtration steps with decreasing pore size filters (150 µm, 100 µm, 50 µm and 30 µm, Sysmex-Partec, Germany). To pellet the nuclei, the 30 µm filtrate was centrifuged for 5 minutes at 4000 g and the pellet washed with cytoplasmic extraction buffer without 0.1% Triton X-100 (adapted from^51^). The pellet was then resuspended in 100 µL of nuclei extraction buffer (50 mM HEPES pH 5.7, 420 mM NaCl, 0.5 mM EDTANa_2_, 0.1 mM EGTA, 10% glycerol) and sonicated for 30 seconds (Bioruptor® Plus Sonication System, Diagenode, Belgium). After incubation on ice for 30 minutes, the homogenate was centrifuged at 11000 g for 10 minutes and the pellet was discarded. Total soluble protein was determined using the Bradford Protein Assay (Bio-Rad, USA) following the manufacturer’s instructions. Nuclear extractions were stored at −80 °C until use.

### Histone deacetylase (HDAC) activity assay

The inhibition of HDACs activity by 2.5 µM SAHA was assessed with EpiQuick HDAC Activity/inhibition Assay Kit (Epigentek, USA), according to the manufacturer’s instructions. Briefly, nuclear extracts, prepared as described above, were incubated for 90 minutes, at 37 °C, with a HDAC specific substrate, in the presence or absence of SAHA. Capture antibody was added and incubated for one hour at room temperature. Then, detection antibody was added and incubated at room temperature for 30 minutes. Technical triplicates were analyzed. Total HDAC inhibition was determined by reading the absorbance at 450 and 655 nm and calculated using the following formula (equation (1)):

1 $$(1-\frac{Inhibitor\,Sample\,OD-Blank\,OD}{No\,Inhibitor\,Sample-Blank\,OD})\times 100 \% $$

### RNA isolation from Medicago cell samples

Total RNA was isolated from cell samples, with and without 2.5 µM SAHA treatment, recovered at days 3, 5, 7 and 11 of the cell growth, with biological triplicates. Cell samples were obtained by vacuum filtration, flash-frozen in liquid nitrogen and stored at −80 °C until RNA isolation was performed. Cell samples were ground with eppendorf pestles in the presence of 500 µL of TRIzol reagent (NZYTech, Portugal). Next, Direct-zol^TM^ RNA MiniPrep (Zymo Research, USA) was used to perform RNA isolation as indicated by manufacturer’s instructions. RNA sample concentrations were measured in a NanoDrop 2000c UV-Vis Spectrophotometer (Thermo Scientific, USA) and run in a 0.8% agarose gel to check for RNA integrity. Genomic DNA contamination in RNA samples was assessed by performing a PCR with primers designed for promoter and terminator of L-PGDS gene. RNA samples were negative for genomic DNA contamination. Samples were flash-frozen in liquid nitrogen and stored at −80 °C until use.

### cDNA synthesis

500 ng of isolated RNA was used to synthesize cDNA using the ImProm-II^TM^ Reverse Transcription System (Promega, USA), according to the manufacturer’s instructions. 0.5 µg of oligo(dT) and random primers (Promega, USA) were used for each reaction. Briefly, RNA and primers were mixed and incubated at 70 °C for 5 minutes and placed on ice for a further 5 minutes. Then, the master mix was added to the previous reaction, to a final volume of 20 µL. The reactions were transferred to a thermocycler (Mastercycler, Eppendorf, Germany) and incubated as follows: 25 °C for 5 minutes, 42 °C for one hour, 70 °C for 15 minutes and then on hold at 4 °C. cDNA samples were stored at −20 °C until use.

### QuantStudio^TM^ 3D Digital PCR

The QuantStudio^TM^ 3D Digital PCR (QS3D; Applied Biosystems, ThermoFischer Scientific, USA) was used to quantify L-PGDS gene expression in the cell samples in the presence and absence of 2.5 µM SAHA. 0.005 ng to 0.25 ng of cDNA were used (so that final copies/µL value for each sample was within the range of 200 and 2000, as recommended by the manufacturer) and a TaqMan® probe (Hs00168748_m1 for human L-PGDS, ThermoFischer Scientific, USA) labeled with FAM was added to the QS3D master mix. Briefly, each QS3D chip was loaded with 14.5 µL of reaction (one chip per replicate) and sealed using a QS3D Chip Loader (ThermoFischer Scientific, USA), following the manufacturer’s instructions. The QS3D chips were loaded into a Dual Flat Bloc GeneAmp^TM^ PCR System 9700 thermal cycler to perform amplification, with the following conditions: 96 °C for 10 minutes, 98 °C for 30 seconds and 60 °C for 2 minutes (both for 40 cycles) and hold at 25 °C (to avoid chip condensation). After amplification, chips were imaged on QS3D Instrument (ThermoFischer Scientific, USA) and data analysis was carried out in QS3D Analysis Suite^TM^ Software, which takes in account the samples dilution factor. The software applies a quantification algorithm based on the Poisson model and estimates copy number/µL mean values with a 95% confidence interval.

### Statistical data analysis

All results obtained from cell viability, from relative quantification of proteins (L-PGDS, acetylated histone H3 and histone H3) and from the histone deacetylase activity assay were statistically analyzed by multiple t-tests (multiple comparisons using the Holm-Šídák method) with α = 0.05 (significance level). All statistical analysis was performed with GraphPad Prism version 6.0c for MacOS^52^. Statistical significance was assumed for p values ≤0.05.

## Electronic supplementary material

Supplementary Information
