# Supplementary material for: Addition of a histone deacetylase inhibitor increases recombinant protein expression in Medicago truncatula cell cultures
Source: Sci Rep. 2017 Dec 1;7:16756. doi: 10.1038/s41598-017-17006-9 (PMC5711867; doi:10.1038/s41598-017-17006-9)
Supplement: Supplementary file 1 — Supplementary Information [file 41598_2017_17006_MOESM1_ESM.pdf]

Addition of a histone deacetylase inhibitor increases recombinant protein expression in *Medicago truncatula* cell cultures

Rita B. Santos, Ana Sofia Pires, Rita Abranches*
Plant Cell Biology Laboratory, Instituto de Tecnologia Química e Biológica António Xavier (ITQB NOVA), Av República, 2780-157 Oeiras, Portugal
*Corresponding Author: Rita Abranches (ritaa@itqb.unl.pt)

Supplementary Information

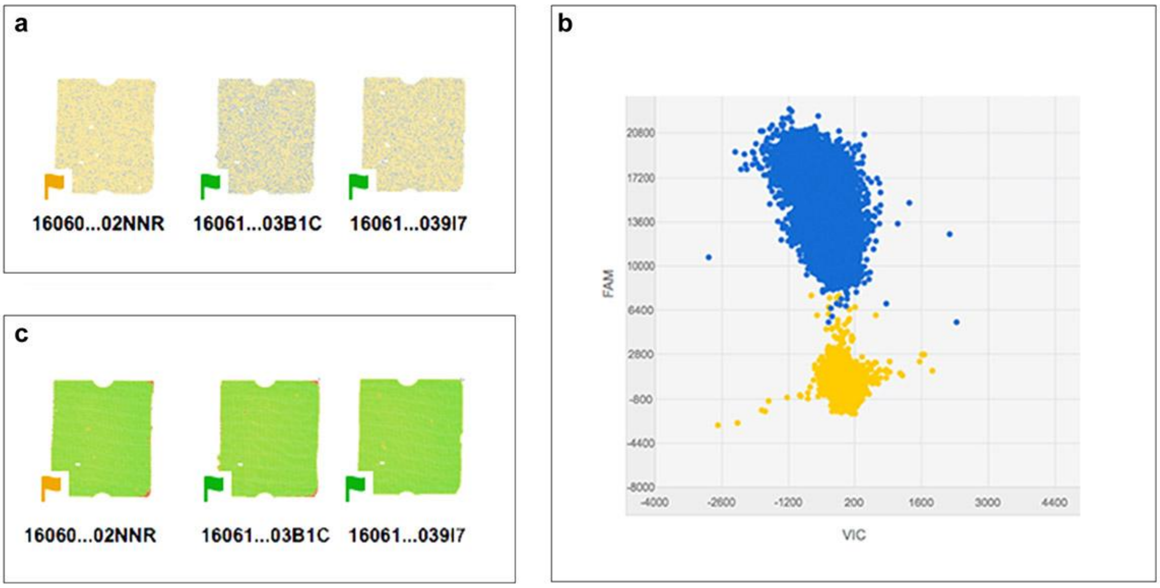

Supplementary Figure 1 – Representative QS3D chip imaging and scatter plot of three biological replicates. (A) Chip view with analysis of PCR reactions (blue dots: FAM signal, yellow dots: no amplification). (B) Chip view with analysis of chip quality (green dots: good quality loading; yellow and red dots: condensation or wrong loading; white dots: automatically filtered dots by software. (C) Scatter plot view of merged biological replicates.

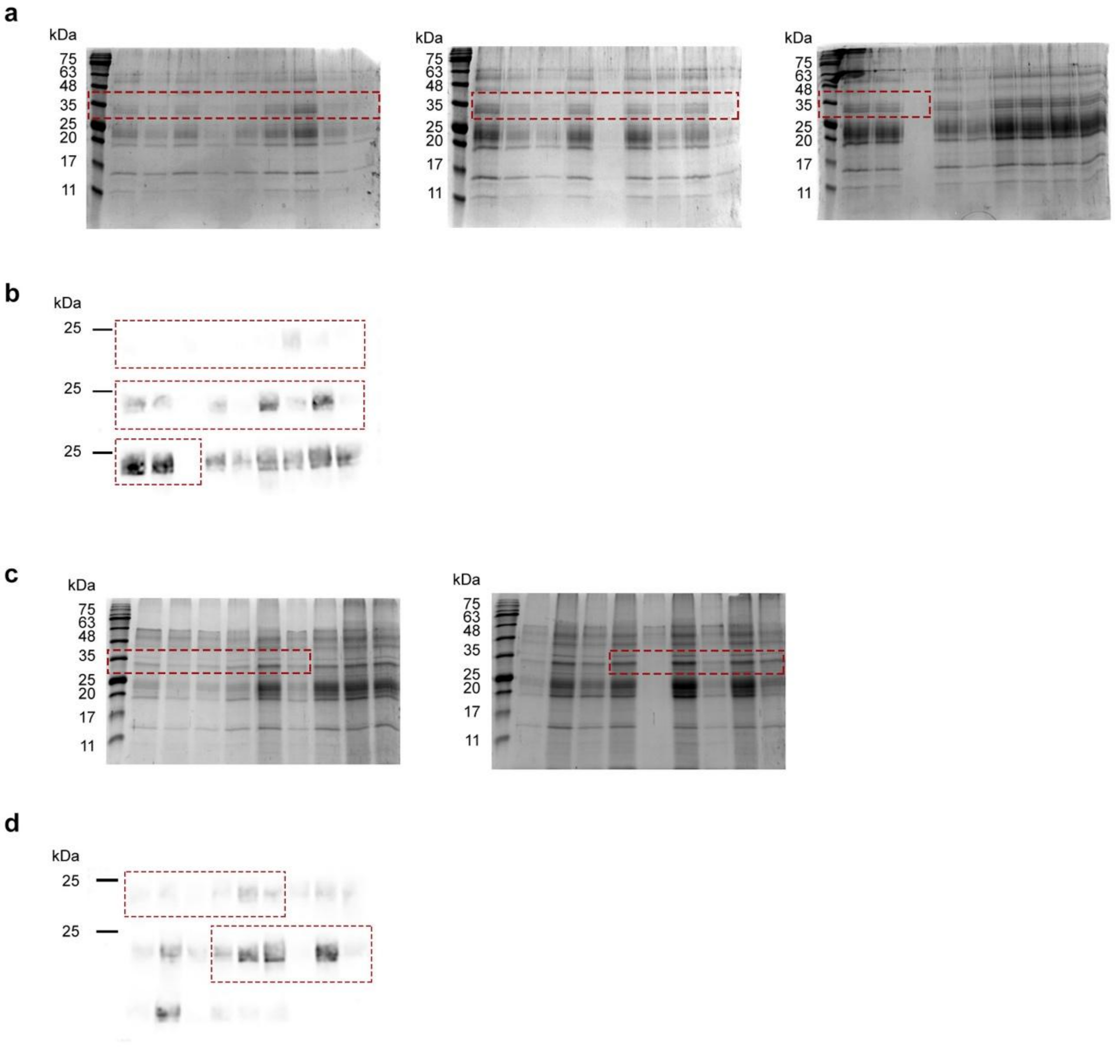

Supplementary Figure 2 – Full-length images of gels and blots for Figure 3. SDS-PAGE gels (a and c) and immunoblots (b and d).

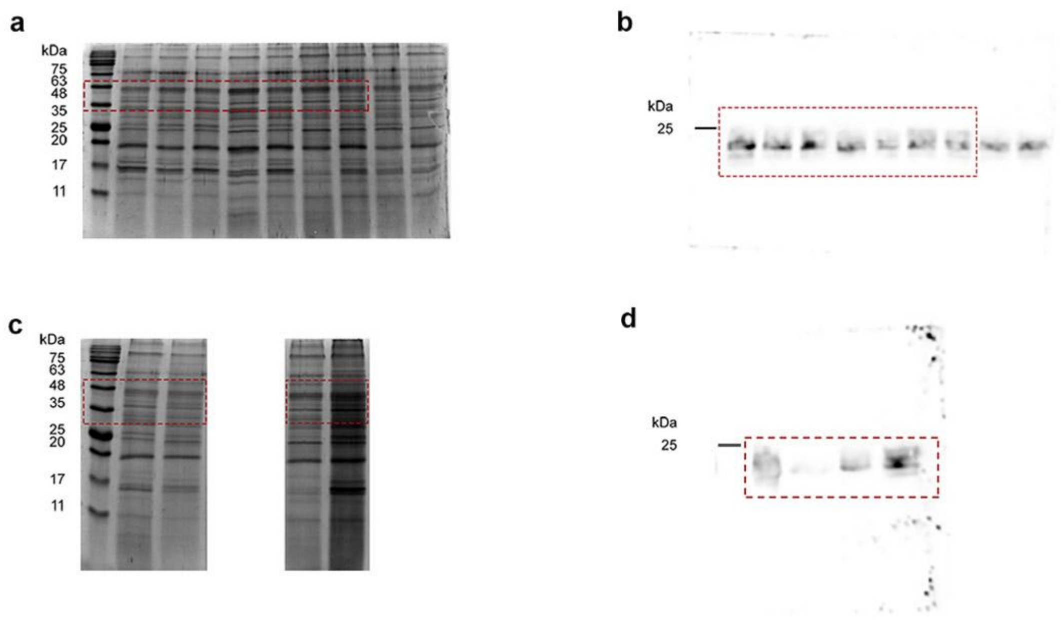

Supplementary Figure 3 – Full-length images of gels and blots for Figure 4. SDS-PAGE gels (a and c) and immunoblots (b and d).

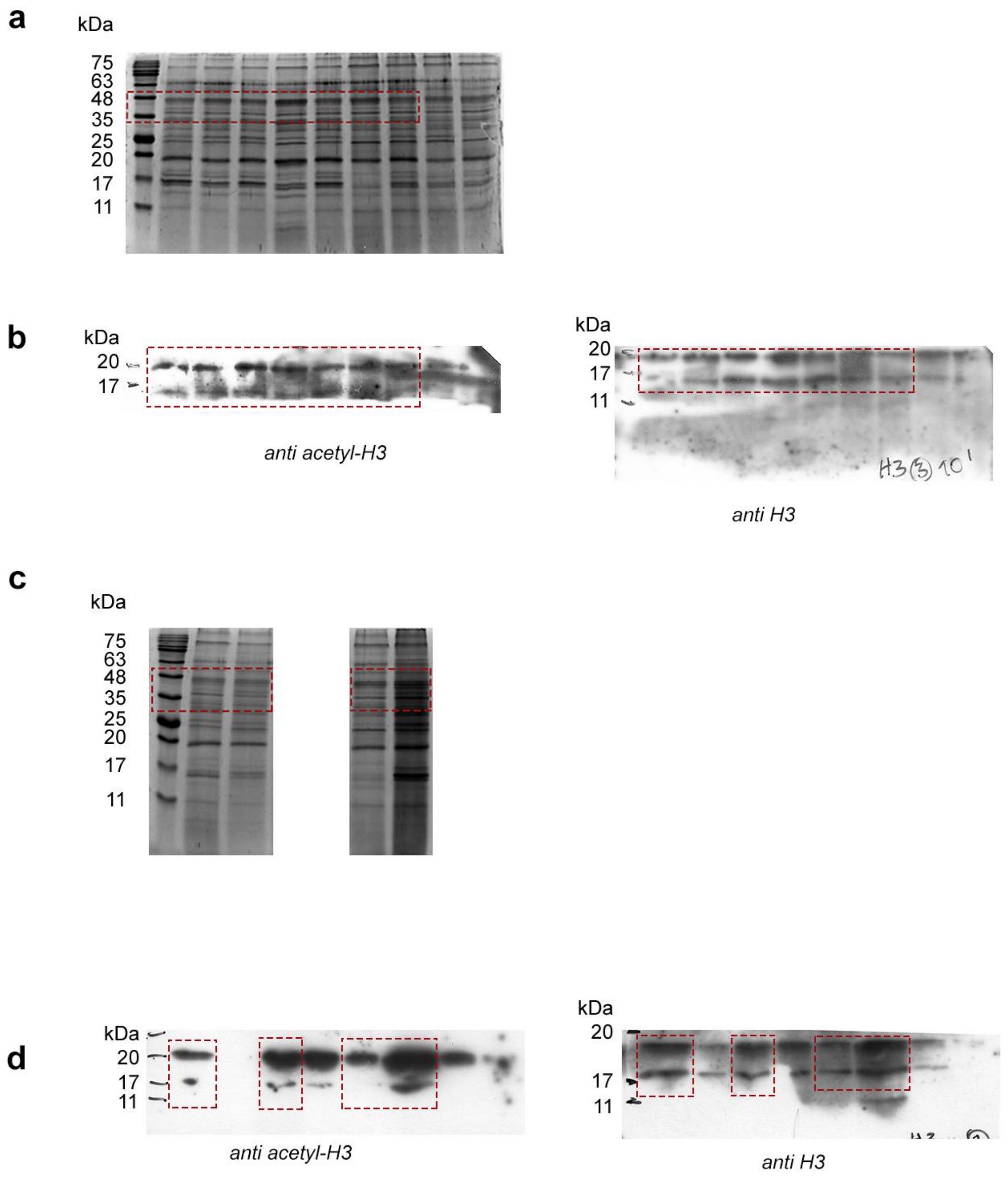

Supplementary Figure 4 – Full-length images of gels and blots for Figure 6. SDS-PAGE gels (a and c, the same gels as figure S2) and immunoblots (b and d).
